# Systemic Protein Biomarkers, Composite Blood Inflammatory Indices and Cellular Ratios in Metastatic Colorectal Cancer: Potential Therapeutic Targets

**DOI:** 10.3390/diseases14050153

**Published:** 2026-04-27

**Authors:** Teresa Smit, Ronald Anderson, Helen C. Steel, Theresa M. Rossouw, Bernardo L. Rapoport

**Affiliations:** 1 The Clinical and Translational Research Unit (CTRU) of the Medical Oncology Centre of Rosebank, Saxonwold, Johannesburg 2196, South Africa; tsmit@rosebankoncology-ctru.co.za (T.S.); ronald.anderson@rosebankoncology-ctru.co.za (R.A.); 2 Department of Immunology, Faculty of Health Sciences, University of Pretoria, Prinshof, Pretoria 0083, South Africa; helen.steel@up.ac.za (H.C.S.); theresa.rossouw@up.ac.za (T.M.R.); 3 Centre for Innovative Immunology Research and Education (IINSPIRE), Department of Paediatrics and Child Health, Faculty of Health Sciences, University of Pretoria, Prinshof, Pretoria 0083, South Africa

**Keywords:** C-reactive protein (CRP), hierarchical cluster analysis, inflammation, interleukin-1β (IL-1β), interleukin-6 (IL-6), interleukin-13 (IL-13), metastatic colorectal cancer, neutrophil/lymphocyte ratio (NLR), therapeutic monoclonal antibody targeting, transforming growth factor-β1 (TGF-β1)

## Abstract

Background/objectives: Although informative, current insights into the inflammatory nature of colorectal cancer (CRC) have yet to have a meaningful impact on the prevention of, and development of novel therapies for, the treatment of this prevalent and challenging disease. Accordingly, the current study was focused on identifying putative, key, systemic, mostly pro-inflammatory biomarkers of metastatic CRC (mCRC) prognosis and outcome. Methods: Patients with mCRC (*n* = 38) and matched healthy controls (*n* = 30) were recruited to the study. A multiplex magnetic bead array system and an ELISA procedure were used to measure the plasma concentrations of selected cytokines (*n* = 25) and that of C-reactive protein (CRP) by immunonephelometry. Systemic inflammatory indices (*n* = 5) were derived from the hematological data. Results: Plasma levels of 17/25 of the cytokine biomarkers and CRP were found to be significantly elevated, while the neutrophil/lymphocyte ratio proved to be the most useful of the various inflammatory indices. Subgroup analysis of the data derived from the group of mCRC patients revealed that the intensity of the systemic inflammatory response was mostly unaffected by tumor location, age, gender, and treatment line. The exception was time to progression, with a shorter time (<120 days) being associated with increased levels of IL-6, IL-8 and TNF-α. Hierarchical cluster analysis of the data revealed a possible association with a small group of four cytokines, comprising IL-1β, IL-13, IL-6/CRP and TGF-β1. Conclusions: This study confirms a strong association of established mCRC with cytokine-driven systemic inflammation. Four of these cytokines, IL-1β/IL-13 IL-6/CRP, and TGF-β1, appear prominent and are possibly indicative of novel targetable therapeutic options.

## 1. Introduction

Almost two million new cases of colorectal cancer (CRC) were estimated globally in 2020, accounting for approximately 9.5 per 100,000 person-years [1]. Notably, there is a significant regional variation, with the highest incidence in Europe, Oceania, and North America, and the lowest in the African and Eastern Mediterranean regions [1]. If current trends continue, the number of new cases of CRC is expected to increase substantially by 2040 [1].

Inflammation plays a crucial role in the development, progression, and prognosis of malignant diseases [2], with chronic inflammation representing both a significant risk factor and a key biological driver associated with colorectal carcinogenesis [3]. In this context, inflammatory bowel diseases, particularly ulcerative colitis and Crohn’s disease, significantly increase the risk of CRC, with those individuals afflicted with a >10-year history of ulcerative colitis having a 5- to 10-fold increased CRC risk [4].

The nuclear factor kappa-light-chain-enhancer of activated B-cells (NF-κB) transcription pathway is a master regulator of inflammation and immune responses. NF-κB activates the production of several pro-inflammatory cytokines, including interleukin (IL)-6 and tumor necrosis factor-alpha (TNF-α), as well as chemokines, which stimulate tumor growth and survival, angiogenesis, and metastasis [5]. In the tumor microenvironment (TME), NF-κB not only drives recruitment and activation of various types of immune suppressive cells, including regulatory T-cells, myeloid-derived suppressor cells (MDSCs), and cancer-associated fibroblasts (CAFs), but also promotes angiogenesis and extracellular matrix remodeling, making the environment favorable for tumor growth and metastasis [6].

This contention is supported by an abundance of supporting evidence describing elevated systemic levels of pro-inflammatory proteins, such as the cytokines/chemokines, IL-1β, IL-6, IL-8, IL-11, IL-17A, IL-22, and TNF-α, and the acute phase reactant, C-reactive protein (CRP), in patients with both early and advanced CRC, which are associated with a poor prognosis [7].

Measurement of the ratios of various types of circulating leukocytes (neutrophils, lymphocytes, monocytes) and platelets calculated from full blood counts (FBCs) have also been used as biomarkers associated with poor outcomes in CRC. These ratios include the neutrophil-to-lymphocyte ratio (NLR), the monocyte-to-lymphocyte ratio (MLR) and the platelet-to-lymphocyte ratio (PLR). In addition, these blood cell counts can be used to calculate systemic inflammatory scores. These include the systemic immune–inflammation index [SII; based on (neutrophils × platelets)/lymphocytes]; the systemic inflammation response index [SIRI; based on (neutrophils × monocytes)/lymphocytes], and the aggregate index of systemic inflammation [AISI; based on (neutrophils × platelets × monocytes)/lymphocytes] [8,9,10,11,12,13].

The objectives of the current study were: (i) to measure and compare the plasma concentrations, correlations and clusters of a range of prominent, predominantly pro-inflammatory cytokines (*n* = 15), chemokines (*n* = 5), cytokine growth factors (*n* = 5), and CRP, as well as inflammatory cellular ratios/indices (*n* = 5), in patients with advanced metastatic CRC (mCRC) relative to those of matched, healthy control participants; (ii) to compare the levels of these systemic biomarkers in the context of the age and gender of the patients, as well as with the location of the primary tumor and line of treatment, and (iii) to identify those biomarkers potentially indicative of disease outcome and early progression (<120 days), as well as their targetable therapeutic potential.

## 2. Patients and Biomarkers

### 2.1. Patients

Patients with advanced CRC (*n* = 38) were recruited to the study together with 30 healthy control participants. Patients with uncontrolled co-morbidities such as diabetes, obesity and cardiovascular disease or current history of any major inflammatory condition, therapy or abnormality that might confound the results of the study were excluded from participation. The median age of the patients was 63 years (range, 27–84). There were 23 males (61%) and 15 females (39%). The primary tumor was localized in the left colon in 21 patients (55%), in the right colon in four patients (11%), and in the rectum in 13 patients (34%). Nineteen patients (50%) had a single metastatic site, while 19 patients (50%) had more than one metastatic site. Eighteen patients (47%) received systemic chemotherapy in the frontline setting, and 20 (53%) patients received systemic chemotherapy in the second-line setting or beyond. Mixed-matched repair, *KRAS* (Kirsten rat sarcoma viral oncogene homolog) and *BRAF* (B-type rapidly accelerated fibrosarcoma neuroblastoma RAS viral oncogene homolog) status data were available on a limited number of patients. Mixed-matched DNA repair proficiency was documented in 22 patients (58%); none were mixed-matched repair-deficient, and 16 (42%) had an unknown mixed-matched repair status. *BRAF* was wild type in 12 patients (31%), mutated in 1 patient (3%), and unknown in 25 patients (66%). *KRAS* status was wild type in 6 patients (16%), mutated in 8 patients (21%), and unknown in 24 patients (63%). *NRAS* (neuroblastoma *RAS* viral oncogene homolog) status was wild type in 13 patients (34%), mutated in 1 patient (3%), and unknown in 24 patients (63%). Treatment consisted of systemic chemotherapy, including oxaliplatin, irinotecan, and/or fluorouracil-based treatments, with or without anti-epidermal growth factor receptor or anti-angiogenesis treatment. Ethics approval was granted by the Research Ethics Committee of the Faculty of Health Sciences, the University of Pretoria (Ethics Committee Approval Number: 854/2020).

### 2.2. Biomarkers

Using the procedures described below, plasma levels of cytokines, chemokines, growth factors, and CRP were measured to identify novel biomarker signatures in this disease. The cytokines included IL-1β, -2, -4, -5, -6, -7, -8, -9, -10, -12, -13, and -17A, TNF-α, interferon gamma (IFN-γ), and the IL-1 receptor antagonist (IL-1Ra). The chemokines measured included monocyte chemoattractant protein-1 (MCP-1; also known as CCL2), macrophage inflammatory protein (MIP)-1α (also known as CCL3), MIP-1β (also known as CCL4), eotaxin (also known as CCL11), and interferon gamma-induced protein-10 (IP-10; also known as CXCL10). The cytokine growth factors measured included fibroblast growth factor (FGF)-basic, granulocyte colony-stimulating factor (G-CSF), granulocyte-macrophage colony-stimulating factor (GM-CSF), platelet-derived growth factor 2 b subunits (PDGF-bb), and vascular endothelial growth factor (VEGF). These biomarkers are representative of a range of cell types involved in chronic inflammatory responses.

Additional pro-inflammatory biomarkers included the inflammatory FBC composite ratios, including the NLR, PLR, and MLR, as well as the SIRI and AISI scores.

## 3. Laboratory Methods

### 3.1. Sample Collection and Processing

Following signed informed consent, blood samples (10 mL) were collected in ethylenediaminetetraacetic acid-containing vacutainers (Becton Dickinson and Co., Franklin Lakes, NJ, USA) by a trained phlebotomist. The blood samples were processed within four hours and the plasma fraction was aliquoted and stored at −80 °C until use.

### 3.2. Biomarker Measurements

Using the procedures described below, plasma levels of cytokines, chemokines, growth factors, and CRP were determined.

#### 3.2.1. Measurement of Circulating Leukocytes/Platelets

The MLR, NLR and PLR were calculated using the results from routine peripheral blood tests, including full blood, neutrophil, platelet, and lymphocyte counts.

#### 3.2.2. Measurement of Systemic Cytokines, Chemokines and Growth Factors

A Bio-Plex Pro Human Cytokine Assay (Bio-Rad Laboratories, Inc., Hercules, CA, USA) was used for determining the circulating levels of cytokines, chemokines and growth factors in the stored plasma samples. The methodology was followed as outlined by the manufacturer and described briefly below.

Magnetic beads (50 μL) were added to each well of a 96-well microplate. Plasma samples diluted four-fold, standards of known concentration, and controls (50 μL) were added to the appropriately designated wells and the microplate was sealed and incubated protected from light at room temperature (22 °C) for 45 min on an orbital plate shaker set to 800 rpm (Thomas Scientific, Swedesboro, NJ, USA). Following the incubation step, the plate was washed three times using an automated magnetic wash station (Bio-Rad Laboratories, Inc.). Detection antibody (25 μL) was added to each well, the microplate was sealed and incubated for an additional 30 min, as described above. The microplate was washed three times and streptavidin-phycoerythrin (50 μL) was added to each well. The microplate was sealed again and incubated for a final 15 min period at room temperature, as described above. The plate was then washed three times using the automated magnetic wash station. The beads were resuspended in 125 μL assay buffer and shaken vigorously for two minutes on a Cooke AM69 microplate shaker (Dynatech AG, Bleichestrasse, Zug, CH) prior to being assayed using a Bio-Plex Suspension Array platform (Bio-Rad Laboratories, Inc.), and Bio-Plex Manager Software 6.0 was used for bead acquisition and analysis of median fluorescence intensity. The results are presented as pg/mL.

#### 3.2.3. Detection of Transforming Growth Factor-β1

Levels of TGF-β1 were determined using an enzyme-linked immunosorbent assay (ELISA) (Elabscience, Houston, TX, USA). Prior to analysis, latent TGF-β1 was activated to the immunoreactive form by adding 40 μL 1 N HCL to 240 μL plasma (diluted 8-fold). After thorough mixing, the acidified sample was incubated for 10 min at room temperature. Following the incubation period, the sample was neutralized by the addition of 50 μL 1.2 N NaOH and mixed thoroughly. The assay was performed immediately according to the manufacturer’s instructions.

The samples (100 µL), at a final 10-fold dilution, were added to the designated wells of a 96-well plate. The plate was incubated for 90 min at 37 °C. The contents of each well were aspirated and biotinylated detection antibody (100 μL) was added immediately followed by further incubation of the pate for 60 min at 37 °C. Following this incubation step, the plate was washed three times using an automated microplate wash station (BioTek Instruments, Inc., Winooski, VT, USA). Horseradish peroxidase conjugate (100 μL) was then added to each well and the plate was sealed and incubated for 30 min at 37 °C. The plate was washed five times, as described above, followed by the addition of substrate reagent (90 μL) to each well. The plate was incubated at 37 °C for 15 min whereafter the color development was stopped by the addition of stop solution (50 μL). The optical density of each well was determined using a PowerWaveX spectrophotometer (BioTek Instruments, Inc.) set to a wavelength of 450 nm and the final results are expressed as pg/mL.

#### 3.2.4. Measurement of C-Reactive Protein Levels

Plasma concentrations of CRP were measured according to the manufacturer’s specifications using a high-sensitivity Atellica NEPH 630 nephelometer (Siemens Healthcare Diagnostics, Newark, NJ, USA). Plasma samples were diluted 20-fold and 150 µL of each sample was aliquoted into the appropriate tubes and placed into the nephelometer. The samples were mixed with polystyrene particles coated with monoclonal antibodies reactive with human CRP. A reference was generated by multi-point calibration, and N Rheumatology Standard SL serial dilutions were automatically prepared by the nephelometer. The results are expressed as mg/L plasma.

## 4. Expression and Statistical Analysis of Results

The primary hypothesis was that there is a significant difference in the plasma levels of the systemic biomarkers, predominantly proinflammatory biomarkers, between patients with mCRC and the healthy controls. Descriptive statistics were used to tabulate patient characteristics. The Shapiro–Wilk test was used to test for normality. Most variables tested were not normally distributed; therefore, nonparametric statistics were used (see Supplementary Tables S1 and S2 for mCRC patients and the healthy controls, respectively). The Mann–Whitney U test with a Bonferroni correction for 10,000 comparisons was used to compare levels of the test biomarkers between mCRC patients and healthy controls. Multiple testing correction was performed using the Benjamini–Hochberg procedure to control for the false discovery rate (FDR) at 0.05 [14].

A correlation matrix report was used to identify correlations between variables (or subsets of variables) within the subset, using the Pearson test to define significance. A *p*-value of <0.05 was considered statistically significant. Time to progression (TTP) was defined as the time from patient enrolment to the first documented progression or death from any cause, whichever occurred first. For patients who did not progress, the TTP was calculated from the date last seen minus the date of registration. Hierarchical cluster analysis (HCA) was performed to explore cytokine expression patterns in plasma samples obtained from patients with mCRC and the healthy controls. Cytokine quantification included pro-inflammatory, anti-inflammatory, chemotactic, and angiogenic mediators. Before analysis, data were Log2-transformed and standardized to ensure comparability across variables. A heatmap was constructed using correlation coefficients for the variables (ranging from −1 to 1). HCA was conducted using the NCSS 2026 statistical software (NCSS LLC, Kaysville, UT, USA). The Group Average [Unweighted Pair-Group Method with Arithmetic Mean (UPGMA)] clustering method was applied using the Euclidean distance metric to measure similarity between variables. Separate dendrograms were generated for the mCRC and control groups to identify clusters of cytokines with correlated expression patterns. Clusters were defined based on visual inspection of the dendrograms. A second HCA was performed to compare patients with a TTP of 120 days or more to those who progressed in less than 120 days. The TTP value of 120 days was selected on the basis of this being the median TTP of the current study cohort, as determined by Kaplan–Meier estimation. The NCSS 2026 software for Windows [NCSS, LLC. (2026). NCSS 2026 Statistical Software (Version 26.0.1). NCSS, LLC. https://www.ncss.com, accessed on 22 April 2026] was used for all statistical analyses.

## 5. Results

### 5.1. Demographic and Clinical Characteristics

The demographic and clinical profiles of the 38 patients with mCRC are shown in Table 1.

### 5.2. Comparison of the Plasma Concentrations of Cytokines, Chemokines, Growth Factors, and CRP in Patients with Metastatic Colorectal Cancer and the Control Participants

These results are shown in Table 2. The plasma concentrations of IL-1β, IL-6, IL-8, IL-10, IL-12, IL-13, IL-17A, CCL11, G-CSF, GM-GSF, IFN-γ, CXCL10, CCL3, CCL4, TNF-α, VEGF and CRP were significantly elevated in the cohort of mCRC patients relative to those of the group of healthy controls (*p* ≤ 0.0432–*p* ≤ 0.0000).

### 5.3. Correction for Multiple Comparisons Between the Various Plasma Test Biomarkers Using the Benjamini–Hochberg False Discovery Rate Procedure

The *p*-values were adjusted for multiple testing using the Benjamini–Hochberg procedure to control for the false discovery rate (FDR). These, together with the Log2-fold changes (Log2-FC), are included in Table 3.

### 5.4. Composite Blood Inflammatory Indices and Cellular Ratios in Metastatic Colorectal Cancer

The median and 95% confidence intervals for AISI, MLR, NLR, PLR and SIRI, are shown in Table 4.

### 5.5. Comparison of the Concentrations of the Test Biomarkers According to Primary Tumor Location in Patients with Metastatic Colorectal Cancer

#### 5.5.1. Primary Colon Location vs. Primary Rectum Location

These results are shown in Supplementary Table S3. Except for NLR (colon cancer 2.30 vs. rectum 3.72, *p*-value = 0.0095), there were no significant differences in the plasma concentrations of CRP, cytokines, chemokines, growth factors, as well as between composite inflammatory indices, MLR, NLR and PLR, between patients with primary colon vs. primary rectal cancer.

#### 5.5.2. First-Line vs. More than One Line of Treatment

These results are shown in Supplementary Table S4. Interestingly, there were no significant differences in the plasma concentrations of cytokines, chemokines, growth factors, CRP, or composite inflammatory indices, MLR, NLR and PLR, between patients with mCRC receiving first-line treatment and those receiving second-line or beyond. Counter-intuitively, these observations indicate that the relationship between disease progression and inflammation intensity in mCRC is not linear.

#### 5.5.3. Age < 60 Years vs. ≥60 Years

These results are shown in Supplementary Table S5. There were no significant differences in plasma concentrations of cytokines, chemokines, growth factors, CRP and composite inflammatory indices MLR, NLR and PLR, between patients with mCRC younger than 60 years of age and those 60 years of age or older.

#### 5.5.4. Male vs. Female Patients with Metastatic Colorectal Cancer

These results are shown in Supplementary Table S6. There were no significant differences in plasma concentrations of cytokines, chemokines, growth factors, CRP, and composite inflammatory indices, MLR, NLR and PLR, between male and female patients with mCRC. Borderline differences in IL-5, IL-10, G-CSF, GM-CSF and PDGF-bb were noted and are probably of questionable significance.

#### 5.5.5. Time to Progression < 120 Days vs. ≥120 Days

These results are shown in Table 5. The plasma concentrations of IL-1Ra (222.26 vs. 185.29, *p* = 0.0201), IL-6 (6.79 vs. 0.83, *p* = 0.0060), IL-8 (21.52 vs. 9.11, *p* = 0.0370) and TGF-β1 (6.23 vs. 4.58, *p* = 0.0025) were significantly higher in patients with a TTP shorter than 120 days than in those with a TTP longer than 120 days. CXCL10 was numerically different; however, it did not reach statistical significance between the two groups (475.04 vs. 361.13, *p* = 0.0700). There were no significant differences in plasma concentrations of CRP and the other variables.

### 5.6. Correlations Between AISI, MLR, NLR, PLR, SIRI, and the Plasma Test Biomarkers

**Figure 1:**
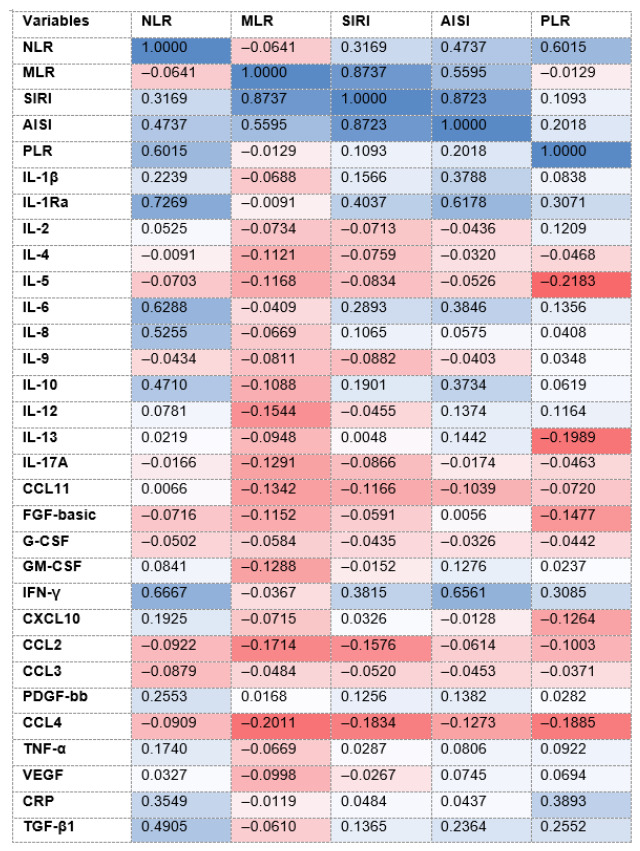
Correlations (r values, blue shading represents maximum value of 1, red shading represents maximum value of −1) between the blood indices and the plasma inflammatory biomarkers. **Abbreviations:** AISI: aggregate index of systemic inflammation; CCL: C–C motif ligand; CRP: C-reactive protein; CXCL: C–X–C motif chemokine ligand; FGF-basic: fibroblast growth factor-basic; G-CSF: granulocyte colony-stimulating factor; GM-CSF: granulocyte-macrophage colony-stimulating factor; IL: interleukin; IFN-γ: interferon gamma; MLR: monocyte-to lymphocyte ratio; NLR: neutrophil-to-lymphocyte ratio; PDGF-bb: platelet-derived growth factor 2 b subunits; PLR: platelet-to-lymphocyte ratio; Ra: receptor antagonist; SIRI: systemic inflammation response index; TGF-β1: transforming growth factor-beta1; TNF-α: tumor necrosis factor-alpha; and VEGF: vascular endothelial growth factor.

As shown in Figure 1, among mCRC patients, moderate-to-strong positive correlations were detected between (1) NLR and PLR, r = 0.6015; IL-1Ra, r = 0.7269; IL-6, r = 0.6288; and IFN-γ, r = 0.6667; (2) MLR and SIRI, r = 0.8737; and (3) SIRI and AISI, r = 0.8723. No significant correlations were detected between either PLR or AISI and the other biomarkers.

### 5.7. Hierarchical Cluster Analysis

#### 5.7.1. Metastatic Colorectal Cancer and the Healthy Controls

**Figure 2:**
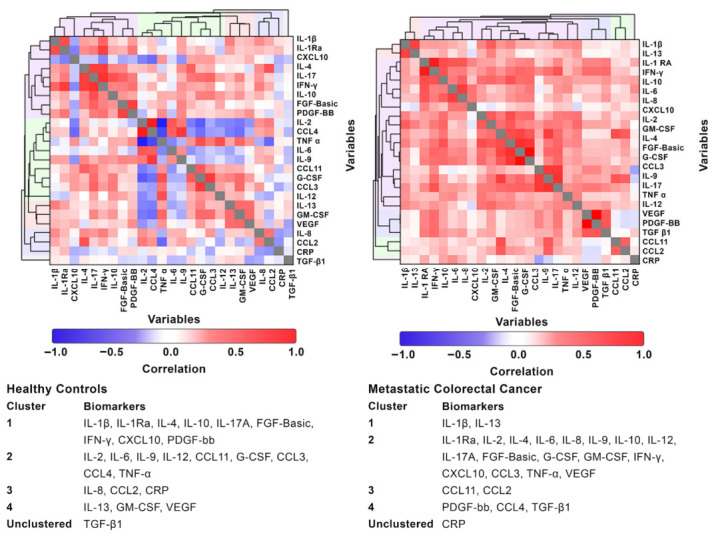
Hierarchical cluster analysis of the healthy controls and patients with metastatic colorectal cancer. **Abbreviations:** CCL: C–C motif ligand; CRP: C-reactive protein; CXCL: C–X–C motif chemokine ligand; FGF-basic: fibroblast growth factor-basic; G-CSF: granulocyte colony-stimulating factor; GM-CSF: granulocyte-macrophage colony-stimulating factor; IL: interleukin; IFN-γ: interferon gamma; PDGF-bb: platelet-derived growth factor 2 b subunits; Ra: receptor antagonist; TGF-β1: transforming growth factor-beta 1; TNF-α: tumor necrosis factor-alpha; and VEGF: vascular endothelial growth factor. Footnote: The heatmaps were constructed using correlation coefficients for the variables (ranging from −1 to 1).

The HCA analysis showed distinct cytokine organization patterns between patients with mCRC and the healthy controls (Figure 2). In healthy individuals, cytokines formed discrete, functionally coherent clusters consistent with homeostatic balanced immune regulation. Pro- and anti-inflammatory mediators (e.g., IL-1β, IL-10, IFN-γ, IL-17A) clustered separately from angiogenic factors (e.g., VEGF, GM-CSF), indicating preserved homeostasis. In contrast, in patients with mCRC there was a collapse of cytokine modularity, characterized by a large, integrated cluster comprising inflammatory (IL-6, TNF-α), regulatory (IL-4, IL-10), and angiogenic (VEGF, FGF-basic) mediators. This cluster reflects systemic immune activation and loss of network compartmentalization, potentially indicating tumor-promoting inflammation. TGF-β1 remained independent in healthy controls; however, it clustered with PDGF-bb and CCL4 in patients with mCRC, which is indicative of enhanced stromal and immunosuppressive interactions. A smaller inflammatory cluster, possibly of significance with respect to disease pathogenesis, comprised of IL-1β and IL-13, was identified. CRP became isolated from cytokine clusters in patients with mCRC, potentially indicating an independent, uncontrolled, systemic, acute-phase response, which requires further validation in a separate, larger cohort.

#### 5.7.2. Metastatic Colorectal Cancer with a TTP < 120 Days Compared with Those with a TTP ≥ 120 Days

**Figure 3:**
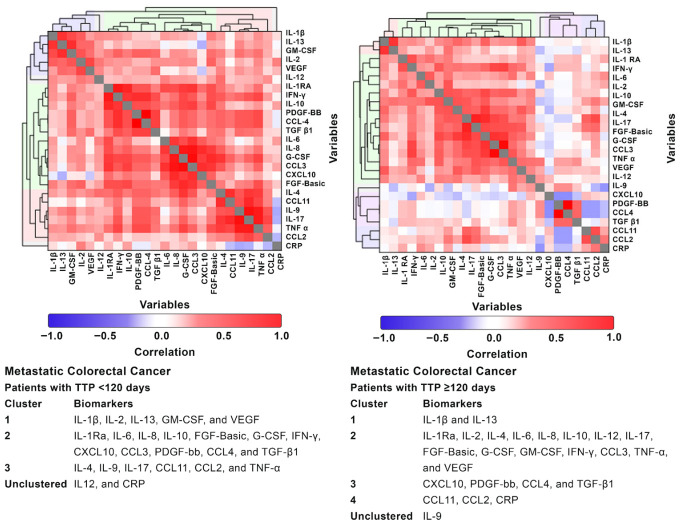
Hierarchical cluster analysis of metastatic colorectal cancer with a time to progression of less than 120 days and 120 days or more than 120 days. **Abbreviations:** CCL: C–C motif ligand; CRP: C-reactive protein; CXCL: C–X–C motif chemokine ligand; FGF-basic: fibroblast growth factor-basic; G-CSF: granulocyte colony-stimulating factor; GM-CSF: granulocyte-macrophage colony-stimulating factor; IL: interleukin; IFN-γ: interferon gamma; PDGF-bb: platelet-derived growth factor 2 b subunits; Ra: receptor antagonist; TGF-β1: transforming growth factor-beta 1; TNF-α: tumor necrosis factor-alpha; and VEGF: vascular endothelial growth factor. Footnote: The heatmaps were constructed using correlation coefficients for the variables (ranging from −1 to 1).

A separate HCA was performed to assess the differences in cytokine organization between patients with mCRC with a short TTP (<120 days) and those with a longer TTP (≥120 days) (Figure 3). Distinct clustering patterns emerged between the two groups, indicating differences in the structure of immune and inflammatory networks associated with disease aggressiveness.

Among patients with a TTP < 120 days, cytokine clustering identified three groups. Cluster 1 included IL-1β, IL-2, IL-13, GM-CSF, and VEGF, combining pro-inflammatory, proliferative, and angiogenic mediators. Cluster 2 grouped IL-1Ra, IL-6, IL-8, IL-10, FGF-basic, G-CSF, IFN-γ, CXCL10, CCL3, PDGF-bb, CCL4, and TGF-β1, representing an extensive inflammatory–regulatory–angiogenic network. Cluster 3 comprised IL-4, IL-9, IL-17A, CCL11, CCL2, and TNF-α; additionally, IL-12 and CRP remained unclustered. Overall, these patients with very aggressive disease exhibit a highly integrated cytokine architecture, with a loss of modular separation among inflammatory, regulatory, and angiogenic signals.

In contrast, patients with a longer TTP (≥120 days) displayed a more structured cytokine profile, similar to that observed in the general mCRC group. Cluster 1 contained IL-1β and IL-13, reflecting localized inflammatory and Th2-type activity. Cluster 2 comprised a large, but organized group—IL-1Ra, IL-2, IL-4, IL-6, IL-8, IL-10, IL-12, IL-17A, FGF-basic, G-CSF, GM-CSF, IFN-γ, CCL3, TNF-α, and VEGF—indicating coordinated, but regulated immune activation. Cluster 3 included CXCL10, PDGF-bb, CCL4, and TGF-β1, suggesting a stromal/angiogenic component, while Cluster 4, with CCL11, CCL2, CRP, and the independent cytokine, IL-9, reflected maintained modularity and separation between immune and acute-phase responses.

## 6. Discussion

Notwithstanding the prognostic utility of blood-based combination inflammatory indices, as well as leukocyte/leukocyte and platelet/leukocyte ratios, detection of increased systemic concentrations of pro-inflammatory cytokines/chemokines, particularly IL-1β, IL-6, IL-8 and TNF-α, represents a prominent, well-researched strategy in CRC/mCRC [15,16,17]. In the prognostic setting of mCRC, these biomarkers are most commonly used in limited combinations, as opposed to individually, with the objective of augmenting predictive potential. Although useful, this strategy does not, however, enable identification of the primary drivers of disease, which is essential for the development of novel, targeted effective therapies.

As a strategy to overcome this disparity, the prognostic value of multiple cytokine/chemokine analyses has been investigated in several studies, focused not only on identification of the most prominent drivers of disease, but also on formulation of a single prognostic score, encompassing multiple cytokines/chemokines. In their systematic review focused on this topic, Gunawardene et al. concluded that “while the data suggests a multi-marker approach may be useful, no real guidance is provided on which markers to use, or how to combine them” [18]. These issues may be overcome by the advent of multi-proteomics technologies, which minimize the inadvertent exclusion of potential key driver biomarkers, with the incorporation of cluster analyses of data and other stringent bioinformatics statistical techniques.

In the current study, the observed notable increases in the systemic concentrations of many of the test inflammatory biomarkers in patients with mCRC relative to the healthy controls are consistent with the existence of a generalized, chronic systemic inflammatory response. Most prominent were significant increases in the plasma concentrations of 9/15 of the test cytokines (IL-1β, -6, -8, -10, -12, -13, -17A, IFN-γ and TNF-α), 4/5 chemokines (CCL3, CCL4, CCL11 and CXCL10) and 4/5 growth factors (G-CSF, GM-CSF, PDGF-bb and VEGF). Sub-group analyses of patients with mCRC revealed that the intensity of the chronic systemic inflammatory response (encompassing cytokines/chemokines/growth factors, systemic inflammatory indices, blood cell ratios and CRP) was unaffected by tumor location, age, first-line of treatment or gender (with the exception of minor increases of IL-5 in females and IL-10, GM-CSF and PDGF-bb in males). Time to progression was a notable exception, however, with a shorter TTP being significantly associated with increased plasma concentrations of three pro-inflammatory, pro-tumorigenic cytokines, namely IL-6, IL-8 and TGF-β1.

With respect to translational potential originating from acquisition of novel insights into disease pathogenesis, hierarchical cluster analysis of cytokine/chemokine comparative data derived from patients with mCRC and the healthy controls revealed significant differences. In the case of the healthy controls, four distinct well-balanced, cytokine clusters of varying composition were detected, which is consistent with a quiescent state of immune system homeostasis. Cluster patterns in patients with mCRC, on the other hand, revealed one extremely large, seemingly dysregulated cluster, populated predominantly by pro-inflammatory/pro-angiogenic cytokines, and three smaller clusters. Of the three smaller clusters, the one comprising IL-1β and IL-13 may be of particular relevance given the probable key involvement of these two cytokines in the initiation and progression stages of CRC. The involvement of IL-1β in CRC, as reflected in the current study by elevated levels of its surrogate biomarker IL-1Ra, tumor cell proliferation, angiogenesis and metastasis, is well recognized [19,20,21,22]. However, less is known about the pro-oncogenic interactions of this cytokine with IL-13.

In this context, several lines of evidence have indeed implicated IL-13 in the pathogenesis of colorectal oncogenesis. In this regard, a German research team, albeit using a preclinical human colonic cell line (HT-29/86), reported that exposure to IL-13 resulted in impairment of epithelial barrier function [23,24]. This damaging effect of IL-13 was due to pro-apoptotic disruption of epithelial tight junctions [23,24]. These findings probably underpin the recent observation that increased systemic levels of the bacterial lipopolysaccharide-binding biomarker, soluble CD14, are associated with risk for development of sporadic CRC [25].

The involvement of IL-13 in the pathogenesis of CRC/mCRC has also been closely linked to the interaction of this cytokine with the high-affinity, alternative IL-13 receptor, interleukin-13 receptor subunit alpha 2 (IL-13RA2). In this context, Barderas et al. in an earlier preclinical study reported high-level expression of IL-13RA2 on the highly metastatic colon cancer cell line, KMI2SM, which was associated with augmentation of “cell adhesion, migration and metastatic colonization” [26]. In the clinical setting, the authors reported that high-level expression of IL-13RA2 was “associated with later stages of disease progression and outcome” [26].

More recently, He et al., working with primary tissue from patients with CRC (*n* = 29) and the CRC cell lines, HCT116 and DLD1, provided insight into the pro-oncogenic mechanisms of IL-13/IL-13RA2 interactions [27]. In addition to confirming significantly upregulated expression of IL-13RA2 on isolated CRC tissue, the authors also observed that this was associated with a stemness phenotype [27]. In a series of experiments using sophisticated technologies, the authors observed that exposure of HCT116 cells to recombinant IL-13 (rIL-13) initiated a series of events, which resulted in polyubiquitination and proteasomal degradation of the key tumor suppressor, p53, and consequent initiation of cancer stem cell tumorigenesis [27]. Confirmatory evidence was derived from a murine model of experimental tumorigenesis, as well as the finding of increased serum levels of IL-13 in patients with CRC. Notably, in the current study, similar to the above findings [27], IL-13 levels were more than four-fold greater in patients with mCRC compared to those of the control cohort.

Taken together, hierarchical clustering analysis of data derived from patients with mCRC and the healthy controls may have identified IL-1β and IL-13 as cytokines, which when operating in a complementary manner, may drive the development and progression of CRC. This may be exacerbated by extensive further dysregulation of other systemic immune/inflammatory mechanisms, which are likely to underpin increases in the levels of other prominent cytokines such as IL-6, IL-8 and TNF-α. In the present study, IL-1β and IL-13 were found to cluster together with IL-2, GM-CSF and VEGF in the patients with a TTP < 120 days.

A separate hierarchical analysis of data was undertaken to identify possible biomarkers that distinguish patients with mCRC who had early progression (<120 days) from those who had disease progression beyond 120 days. This analysis revealed the pro-tumorigenic prominence of TGF-β1 and CRP, as a probable surrogate for IL-6, in the former group, possibly linked to the well-recognized immunosuppressive activities of these two biomarkers [28,29].

With respect to therapeutic implications, IL-6, IL-1β, TGF-β1, and IL-13 (and/or their receptors) are implicated in CRC progression and metastasis; importantly each pathway has druggable targets. These targets include monoclonal antibodies, receptor antagonists, and small-molecule inhibitors at various stages of development, from preclinical proof-of-concept to clinical trials.

Interleukin-6 promotes tumor proliferation, survival, angiogenesis, and STAT3 (Signal Transducer and Activator of Transcription 3) activation. Elevated IL-6 correlates with worse outcomes in CRC in many observational studies. There is a clear therapeutic biological rationale for blocking IL-6; however, clinical single-agent activity in mCRC has so far been limited, suggesting that combination strategies (with chemotherapy, targeted therapy, or immunotherapy) or careful patient selection/biomarkers may be needed. Drugs such as tocilizumab (a recombinant humanized anti-IL-6 receptor monoclonal antibody) have primarily been used in the treatment of COVID-19 complications and rheumatoid arthritis [30,31].

Interleukin-1β is produced primarily by activated macrophages and myeloid cells through the NOD-, LRR- and pyrin domain-containing protein 3 (NLRP3) inflammasome pathway. It promotes angiogenesis, immune suppression (via MDSCs and IL-6 induction), and tumor invasiveness. Canakinumab binds circulating IL-1β with high affinity and prevents its interaction with the IL-1 receptor complex, thereby blocking downstream signaling through the NF-κB and mitogen-activated protein kinase (MAPK) pathways [32].

Canakinumab was investigated in combination with pembrolizumab to target immunosuppression and cancer-related inflammation in patients with non-small cell lung cancer (NSCLC) [33]. The CANOPY-1 was a Phase III, first-line NSCLC study of canakinumab in combination with pembrolizumab and platinum-based chemotherapy, compared to pembrolizumab in combination with chemotherapy. Patients with non-squamous (adenocarcinoma) and squamous histologies were eligible. However, addition of canakinumab did not result in a significant improvement in overall survival or TTP [34].

To our knowledge, there are no Phase III studies targeting IL-1β and IL-6 in patients with mCRC aimed at registering these agents for this disease. Future strategies may require combinations with chemotherapy, with or without immunotherapy, as well as better biomarker selection.

The TME plays a crucial role in carcinogenesis, progression, immune evasion, and metastasis. TGF-β signaling influences the TME, promoting CRC progression. Additionally, increased TGF-β signaling also promotes the conversion of fibroblasts into CAFs, further accelerating disease progression and metastasis [35]. 

Galunisertib targets the TGF-β type I receptor kinase, blocking pro-tumorigenic signals. This agent is undergoing clinical research in the neoadjuvant setting in combination with chemotherapy, with the aim of overcoming drug resistance. The authors hypothesized that adding galunisertib to neoadjuvant chemoradiotherapy would improve pathological complete response (pCR) rates in patients with locally advanced rectal cancer [36]. In a Phase II study, 38 participants were enrolled (NCT02688712). The pCR rate was 32%; however, this was only a Phase II study. Superiority needs to be confirmed in an adequately powered Phase III study.

An expansion cohort of a Phase I trial (NCT02517398) evaluated bintrafusp alfa (a first-in-class bifunctional fusion protein targeting TGF-β and PD-L1) in patients with metastatic microsatellite instability-high (MSI-H) cancers that progressed after immunotherapy. The study aimed to overcome resistance to checkpoint inhibitors and demonstrate that a dual-targeted approach could potentially restore the immune response in heavily pretreated patients with refractory mCRC. Results were disappointing; the study showed an objective response rate of 3.1% and a disease control rate of 6.3% (one partial response, one stable disease) among 32 patients in this cohort [37]. Nevertheless, TGF-β remains an interesting target. Substantial research is required to incorporate this concept into routine clinical practice.

Interleukin-13 works as a crucial driver of progression of mCRC, often acting in synergy with the TGF-β1 pathway. IL-13 is associated with aggressiveness, metastasis, and the maintenance of cancer stem cells [27]. Both cytokines signal through epithelial–mesenchymal transition, recruit normal fibroblasts and convert them into CAFs, which provide a protective environment for the tumor [38]. IL-13RA2 has emerged as a prognostic biomarker for identifying patients at high risk of liver metastasis and disease recurrence [26]. Recently, a novel experimental strategy based on blocking IL-13 tumorigenic activity through immunization with a highly conserved D1 peptide selected from the IL-13RA2 binding site, and on inhibiting cell invasion capacity, has been described [39]. The antibody has highly selective blocking activity against IL-13 and IL-13RA2 and completely inhibits liver metastasis in IL13RA2-positive CRC cells. To our knowledge, however, no active clinical trials are underway using an anti-IL-13RA2 antibody as immunotherapy in CRC; targeting of this receptor should be considered as a priority approach.

Limitations of the current study include the fact that this is a biomarker discovery cohort, a single-institution study with a relatively small sample size. We concede that this may lead to selection bias. These findings should be confirmed in a larger validation cohort in a multi-center setting. In addition, repeated biomarker analysis might provide valuable information regarding the dynamics about their potential value in response to treatment.

## 7. Conclusions

The current study presents novel insights into inflammatory signatures of biomarkers in patients with mCRC. In addition to chemotherapy, administration of monoclonal antibodies that target putative key drivers of inflammation/immunosuppression, particularly IL-1β, IL-6, IL-13 (IL13RA2) and TGF-β1, is warranted. Prospective randomized studies incorporating these agents in the treatment of patients with mCRC should be undertaken to improve the prognosis of this increasingly challenging and ominous disease.

## Figures and Tables

**Table 1:** Patient demographic and clinical profiles.

Demographics (*n* = 38)	
Median Age = 63 Years (Range 27 to 84)	
Gender	
Male	23 (61%)
Female	15 (39%)
Site	
Left colon	21 (55%)
Right colon	4 (11%)
Rectum	13 (34%)
Metastatic sites	
1 metastatic site	19 (50%)
>1 metastatic site	19 (50%)
Line of treatment	
First line	18 (47%)
>1 line	20 (53%)
Mutations	
*MMR*	
Repair-proficient	22 (58%)
Repair-deficient	0 (0%)
Unknown	16 (42%)
*KRAS*	
Wild type	6 (16%)
Mutated	8 (21%)
Unknown	24 (63%)
*BRAF*	
Wild type	12 (31%)
Mutated	1 (3%)
Unknown	25 (66%)
*NRAS*	
Wild type	13 (34%)
Mutated	1 (3%)
Unknown	24 (63%)

Abbreviations: *BRAF:* B-type rapidly accelerated fibrosarcoma; *KRAS*: Kirsten rat sarcoma viral oncogene homolog; *MMR*: mismatch repair; and *NRAS*: neuroblastoma *RAS* viral oncogene homolog.

**Table 2:** Comparison of the plasma levels of cytokines, chemokines, growth factors, and CRP in mCRC patients and the controls.

Variable	Group	Median	95% CI	*p*-Value
CCL2	Control	16.365	13.82–20.61	0.0804
	Patient	19.18	16.86–23.30	
CCL3	Control	1.29	1.02–1.51	**0.0000**
	Patient	2.43	2.13–2.96	
CCL4	Control	154.40	120.36–171.94	**0.0000**
	Patient	469.13	374.68–554.43	
CCL11	Control	38.79	28.88–48.90	**0.0001**
	Patient	60	53.87–63.15	
CRP	Control	1.585	0.415–3.37	**0.0074**
	Patient	4.16	2.90–7.58	
CXCL10	Patient	216.73	183.63–266.06	**0.0001**
	Patient	392.19	303.32–468.89	
FGF-basic	Control	19.86	16.15–22.74	0.4307
	Patient	19.64	19.64–22.79	
G-CSF	Control	66.395	57.71–102.43	**0.0000**
	Patient	155.28	135.86–185.13	
GM-CSF	Control	0.76	0.56–0.81	**0.0000**
	Patient	3.04	2.67–3.74	
IFN-γ	Control	6.83	6.13–7.27	**0.0386**
	Patient	8.31	6.56–10.03	
IL-1β	Control	1.025	0.77–1.41	**0.0089**
	Patient	2.08	1.20–2.63	
IL-1Ra	Control	233.53	146.83–339.07	0.5951
	Patient	192.98	169.37–229.33	
IL-2	Control	1.63	1.63–1.67	**0.0163**
	Patient	0.25	0.25–1.31	
IL-4	Control	2.77	2.38–3.16	0.1024
	Patient	3.16	2.65–3.65	
IL-5	Control	5.34	5.34–5.86	0.3027
	Patient	8.08	0.93–18.22	
IL-6	Control	0.49	0.14–0.77	**0.0096**
	Patient	2.06	0.61–4.13	
IL-8	Control	3.45	2.29–5.77	**0.0000**
	Patient	12.89	8.00–16.56	
IL-9	Control	367.07	331.34–432.52	0.1096
	Patient	340.09	334.38–362.46	
IL-10	Control	0.900	0.75–1.27	**0.0000**
	Patient	2.305	1.29–3.93	
IL-12	Control	3.00	1.9–8.46	**0.0000**
	Patient	15.26	15.26–21.74	
IL-13	Control	1.685	1.24–2.48	**0.0000**
	Patient	6.98	5.11–8.75	
IL-17A	Control	13.18	11.7–17.36	**0.0000**
	Patient	21.45	19.23–23.11	
PDGF-bb	Control	369.91	135.14–454.78	**0.0033**
	Patient	469.13	374.68–554.43	
TGF-β1	Control	5.56	3.35–9.48	0.6568
	Patient	4.95	4.57–6.06	
TNF-α	Control	55.49	48.62–73.32	**0.0000**
	Patient	134.5	124.12–140.67	
VEGF	Control	12.94	9.1–16.75	0.0875
	Patient	9.1	9.10–70.92	

**Abbreviations:** CCL: C–C motif ligand; CRP: C-reactive protein; CXCL: C–X–C motif chemokine ligand; FGF-basic: fibroblast growth factor-basic; G-CSF: granulocyte colony-stimulating factor; GM-CSF: granulocyte-macrophage colony-stimulating factor; IL: interleukin; IFN-γ: interferon gamma; PDGF-bb: platelet-derived growth factor 2 b subunits; Ra: receptor antagonist; TGF-β1: transforming growth factor-beta 1; TNF-α: tumor necrosis factor-alpha; and VEGF: vascular endothelial growth factor. Values in bold denote significance. Footnote: The Mann–Whitney U test with a Bonferroni correction was used to compare levels of the test biomarkers between mCRC patients and the healthy controls.

**Table 3:** Biomarkers adjusted for Benjamini–Hochberg false discovery rates (FDRs), with an FDR threshold of 0.05.

Biomarker	Mean (Patients)	Mean (Controls)	Log2-FC	*p*-Value	*q*-Value	Discovery
CCL3	6.45	1.30	2.3088	0.0959	0.1312	No
CCL4	102.65	146.09	−0.5092	**0.0000**	**0.0000**	Yes
G-CSF	260.75	77.55	1.7496	**0.0000**	**0.0000**	Yes
GM-CSF	3.42	1.14	1.5864	**0.0001**	**0.0002**	Yes
IL-8	23.21	4.08	2.5068	**0.0033**	**0.0066**	Yes
IL-10	3.21	1.52	1.0797	**0.0010**	**0.0022**	Yes
IL-12	18.53	6.28	1.5604	0.4730	0.5124	No
IL-13	6.96	2.73	1.3484	**0.0000**	**0.0000**	Yes
TNF-α	130.04	73.13	0.8304	**0.0000**	**0.0000**	Yes
IL-17A	21.70	15.54	0.4817	**0.0357**	0.0546	No
CCL11	62.85	40.99	0.6165	**0.0089**	**0.0165**	Yes
CXCL10	594.97	269.48	1.1427	0.5529	0.5750	No
PDGF-bb	510.75	336.01	0.6041	**0.0123**	**0.0213**	Yes
CRP	23.16	1.83	3.6603	0.1457	0.1722	No
IL-1β	2.35	1.17	1.0046	0.3880	0.4386	No
IL-6	8.27	0.75	3.4614	**0.0161**	**0.0262**	Yes
IL-2	1.43	1.24	0.2134	**0.0000**	**0.0000**	Yes
IFN-γ	11.09	7.55	0.5536	0.1080	0.1337	No
MCP1	20.73	16.57	0.3234	**0.0000**	**0.0000**	Yes
VEGF	72.27	22.06	1.7117	**0.0000**	**0.0000**	Yes
IL-4	3.44	2.83	0.2835	**0.0000**	**0.0000**	Yes
IL-9	334.88	374.87	−0.1627	**0.0000**	**0.0000**	Yes
IL-5	18.21	7.07	1.3655	**0.0432**	0.0624	No
FGF-basic	21.94	18.67	0.2332	0.8722	0.8722	No
IL-1Ra	366.42	263.54	0.4755	**0.0000**	**0.0000**	Yes
TGF-β1	5.56	6.96	−0.3245	0.1036	0.1337	No

**Abbreviations:** CCL: C–C motif ligand; CRP: C-reactive protein; CXCL: C–X–C motif chemokine ligand; FGF-basic: fibroblast growth factor-basic; G-CSF: granulocyte colony-stimulating factor; GM-CSF: granulocyte-macrophage colony-stimulating factor; IL: interleukin; IFN-γ: interferon gamma; PDGF-bb: platelet-derived growth factor 2 b subunits; Ra: receptor antagonist; TGF-β1: transforming growth factor-beta1; TNF-α: tumor necrosis factor-alpha; and VEGF: vascular endothelial growth factor. Values in bold denote significance.

**Table 4:** Composite inflammatory indices, leukocyte/leukocyte and leukocyte/platelet ratios as indicators in metastatic colorectal cancer.

	Median	95% CI
AISI	469.69	159.52–966.86
MLR	0.35	0.24–0.64
NLR	2.00	1.36–5.65
PLR	143.00	97.00–301.00
SIRI	1.88	0.74–3.57

Abbreviations: AISI: aggregate index of systemic inflammation; MLR: monocyte-to-lymphocyte ratio; NLR: neutrophil-to-lymphocyte ratio; PLR: platelet-to-lymphocyte ratio; and SIRI: systemic inflammation response index.

**Table 5:** Comparison of the concentrations of the test biomarkers of metastatic colorectal cancer patients with a TTP duration shorter than 120 days and those of 120 days or longer.

Variable	TTP (Days)	Median	95% CI	*p*-Value
CCL2	<120	19.56	14.47–24.03	0.5469
	≥120	18.03	16.47–23.30	
CCL3	<120	2.50	1.60–4.54	0.5806
	≥120	2.21	1.79–2.96	
CCL4	<120	110.15	99.53–114.83	0.2832
	≥120	104.19	99.22–108.02	
CCL11	<120	60.32	44.73–65.98	0.8062
	≥120	58.89	45.05–71.69	
CRP	<120	5.79	1.22–33.60	0.3000
	≥120	3.53	2.51–5.83	
CXCL10	<120	475.04	303.32–737.53	0.0705
	≥120	361.13	226.75–456.83	
FGF-basic	<120	20.45	17.94–25.70	0.8469
	≥120	19.64	16.15–22.79	
G-CSF	<120	157.54	126.51–242.09	0.3905
	≥120	142.78	126.51–185.13	
GM-CSF	<120	3.40	1.84–4.70	0.7691
	≥120	2.67	1.84–3.74	
IFN-γ	<120	9.39	6.56–14.27	0.0734
	≥120	7.44	6.56–8.31	
IL-1β	<120	2.28	0.55–3.80	0.9633
	≥120	2.03	1.41–2.83	
IL-1Ra	<120	222.26	169.37–443.16	**0.0201**
	≥120	185.29	152.63–210.35	
IL-2	<120	0.25	0.25–1.31	0.4681
	≥120	0.25	0.25–1.31	
IL-4	<120	3.04	2.38–3.65	0.2076
	≥120	3.16	2.38–3.88	
IL-5	<120	12.29	0.93–22.01	0.9456
	≥120	0.95	0.93–18.22	
IL-6	<120	6.79	1.66–11.15	**0.0060**
	≥120	0.83	0.12–2.45	
IL-8	<120	21.52	7.72–39.56	**0.0370**
	≥120	9.11	5.13–15.78	
IL-9	<120	346.92	327.52–363.22	0.3665
	≥120	342.75	319.88–368.51	
IL-10	<120	1.97	1.27–6.43	0.6178
	≥120	1.97	1.29–3.93	
IL-12	<120	18.50	11.91–21.74	0.5549
	≥120	15.26	8.46–21.74	
IL-13	<120	6.98	3.04–9.82	0.8901
	≥120	7.44	5.11–9.40	
IL-17A	<120	21.45	16.99–24.75	0.8658
	≥120	20.35	18.12–23.66	
PDGF-bb	<120	504.86	302.43–663.83	0.2763
	≥120	379.59	264.11–554.43	
TGF-β1	<120	6.23	4.83–7.76	**0.0025**
	≥120	4.58	3.50–5.03	
TNF-α	<120	140.16	125.17–146.81	0.1672
	≥120	132.43	115.74–140.67	
VEGF	<120	34.40	9.10–138.95	0.9058
	≥120	9.10	9.10–59.7	
AISI	<120	587.73	261.13–966.86	0.2973
	≥120	426.73	159.52–787.95	
MLR	<120	0.51	0.24–0.64	0.3418
	≥120	0.34	0.25–0.42	
NLR	<120	3.26	1.78–5.65	0.1883
	≥120	2.85	1.36–3.62	
PLR	<120	154.00	117.00–301.00	0.6256
	≥120	142.00	97.00–173.00	
SIRI	<120	2.55	0.92–3.57	0.3116
	≥120	1.79	0.74–2.28	

**Abbreviations:** AISI: aggregate index of systemic inflammation; CCL: C–C motif ligand; CRP: C-reactive protein; CXCL: C–X–C motif chemokine ligand; FGF-basic: fibroblast growth factor-basic; G-CSF: granulocyte colony-stimulating factor; GM-CSF: granulocyte-macrophage colony-stimulating factor; IL: interleukin; IFN-γ: interferon gamma; MLR: monocyte-to lymphocyte ratio; NLR: neutrophil-to-lymphocyte ratio; PDGF-bb: platelet-derived growth factor 2 b subunits; PLR: platelet-to-lymphocyte ratio; Ra: receptor antagonist; SIRI: systemic inflammation response index; TGF-β1: transforming growth factor-beta1; TNF-α: tumor necrosis factor-alpha; and VEGF: vascular endothelial growth factor. Values in bold denote significance.

## Data Availability

The raw data supporting the conclusions of this article will be made available by the authors on request.

## Supplementary Materials

The following supporting information can be downloaded at: https://www.mdpi.com/article/10.3390/diseases14050153/s1, Table S1: Biomarkers tested for normality in metastatic CRC patients using the Shapiro-Wilk test for normality. Table S2: Biomarkers tested for normality in healthy controls using the Shapiro-Wilk test for normality. Table S3: Comparison of the concentrations of test plasma biomarkers, composite inflammatory indices, NLR, MLR and PLR between primary colon vs primary rectum locations. Table S4: Comparison of the concentrations of the test plasma biomarkers, and composite inflammatory indices, NLR, MLR and PLR in metastatic CRC patients receiving first-line treatment vs those with metastatic CRC receiving therapy in the second-line setting or beyond. Table S5: Comparison of the concentrations of plasma test biomarkers and composite inflammatory indices, NLR, MLR, and PLR in metastatic CRC patients younger than sixty years of age and those sixty years of age and older. Table S6:Comparison of the concentration of the plasma test biomarkers, NLR, MLR and PLR in metastatic CRC patients by gender.
