# Supplementary material for: Systemic Protein Biomarkers, Composite Blood Inflammatory Indices and Cellular Ratios in Metastatic Colorectal Cancer: Potential Therapeutic Targets
Source: Diseases. 2026 Apr 27;14(5):153. doi: 10.3390/diseases14050153 (PMC13205856; doi:10.3390/diseases14050153)
Supplement: Supplementary file 1 [file diseases-14-00153-s001.zip › diseases-4217187-supplementary.pdf]

Supplementary Tables

Supplementary Table S1: Biomarkers tested for normality in metastatic colorectal cancer patients using the Shapiro-Wilk test for normality.

Shapiro-Wilk – Metastatic CRC Patients					
**Biomarker**	**Mean**	**Std Dev**	**Test Value**	**Prob Level**	**Decision (Alpha = 5%)**
CCL2	20.73	10.20	0.720	0.0000	Reject normality
CCL3	6.45	20.71	0.226	0.0000	Reject normality
CCL4	102.65	17.45	0.801	0.0000	Reject normality
CCL11	62.85	23.63	0.832	0.0000	Reject normality
CRP	22.67	53.43	0.449	0.0000	Reject normality
CXCL10	594.97	789.28	0.478	0.0000	Reject normality
FGF-basic	21.94	9.31	0.678	0.0000	Reject normality
G-CSF	260.75	519.68	0.282	0.0000	Reject normality
GM-CSF	3.42	1.98	0.884	0.0009	Reject normality
IFN-γ	11.09	9.98	0.591	0.0000	Reject normality
IL-1β	2.35	1.86	0.902	0.0028	Reject normality
IL-1Ra	366.42	505.87	0.499	0.0000	Reject normality
IL-2	1.43	2.56	0.528	0.0000	Reject normality
IL-4	3.43	1.72	0.721	0.0000	Reject normality
IL-5	18.20	28.87	0.636	0.0000	Reject normality
IL-6	8.27	15.849	0.563	0.0000	Reject normality
IL-8	23.21	30.76	0.642	0.0000	Reject normality
IL-9	334.88	57.72	0.759	0.0000	Reject normality
IL-10	3.21	2.41	0.799	0.0000	Reject normality
IL-12	18.53	10.67	0.955	0.1313	Can't reject normality
IL-13	6.96	3.71	0.971	0.4117	Can't reject normality
IL-17A	21.70	6.87	0.887	0.0011	Reject normality
PDGF-bb	510.75	287.92	0.927	0.0157	Reject normality
TGF-β1	5.56	2.12	0.911	0.0054	Reject normality
TNF-α	130.04	25.33	0.908	0.0043	Reject normality
VEGF	72.27	108.66	0.638	0.0000	Reject normality
AISI	1759.94	4809.50	0.354	0.0000	Reject normality
MLR	1.07	3.72	0.218	0.0000	Reject normality
NLR	3.61	2.80	0.837	0.0001	Reject normality
PLR	188.15	133.96	0.773	0.0000	Reject normality
SIRI	4.94	11.20	0.409	0.0000	Reject normality

Abbreviations: AISI: aggregate index of systemic inflammation; CCL: C-C motif ligand; CRP: C-reactive protein; CXCL: C-X-C motif chemokine ligand; FGF-basic: fibroblast growth factor-basic; G-CSF: granulocyte colony-stimulating factor; GM-CSF: granulocyte-macrophage colony-stimulating factor; IL: interleukin; IFN-γ: interferon gamma; MLR: monocyte-to lymphocyte ratio; NLR: neutrophil-to-lymphocyte ratio; PDGF-bb: platelet-derived growth factor 2 b subunits; PLR: platelet-to-lymphocyte ratio; Ra: receptor antagonist; SIRI: systemic inflammation response index; TGF-β1: transforming growth factor-beta1; TNF-α: tumor necrosis factor-alpha; VEGF: vascular endothelial growth factor.

Supplementary Table S2: Biomarkers tested for normality in healthy controls using the Shapiro-Wilk test for normality.

	Shapiro-Wilk – Healthy Controls				
**Biomarker**	**Mean**	**Std Dev**	**Test Value**	**Prob Level**	**Decision (Alpha = 5%)**
CCL2	16.57	5.85	0.977	0.7482	Can't reject normality
CCL3	1.30	0.51	0.938	0.0785	Can't reject normality
CCL4	146.09	48.36	0.940	0.0937	Can't reject normality
CCL11	40.99	17.14	0.950	0.1695	Can't reject normality
CRP	1.83	1.45	0.868	0.0949	Can't reject normality
CXCL10	269.48	146.13	0.847	0.0005	Reject normality
FGF-basic	18.66	9.83	0.962	0.3567	Can't reject normality
G-CSF	77.55	35.50	0.918	0.0235	Reject normality
GM-CSF	1.14	1.32	0.630	0.0000	Reject normality
IFN-γ	7.55	3.60	0.747	0.0000	Reject normality
IL-1β	1.17	0.71	0.954	0.2146	Can't reject normality
IL-1Ra	263.53	33.85	0.909	0.0140	Can't reject normality
IL-2	1.24	0.73	0.731	0.0000	Reject normality
IL-4	2.82	1.34	0.742	0.0000	Reject normality
IL-5	7.06	16.00	0.272	0.0000	Reject normality
IL-6	0.75	0.99	0.670	0.0000	Reject normality
IL-8	4.08	3.00	0.883	0.0033	Reject normality
IL-9	374.87	75.56	0.923	0.0319	Reject normality
IL-10	1.52	2.16	0.389	0.0000	Reject normality
IL-12	6.28	6.05	0.810	0.0001	Reject normality
IL-13	2.73	2.54	0.744	0.0000	Reject normality
IL-17A	15.54	9.25	0.611	0.0000	Reject normality
PDGF-bb	336.01	281.85	0.892	0.0055	Reject normality
TGF-β1	6.96	5.19	0.728	0.0019	Reject normality
TNF-α	73.13	34.66	0.868	0.0015	Reject normality
VEGF	22.06	36.31	0.473	0.0000	Reject normality

Abbreviations: CCL: C-C motif ligand; CRP: C-reactive protein; CXCL: C-X-C motif chemokine ligand; FGF-basic: fibroblast growth factor-basic; G-CSF: granulocyte colony-stimulating factor; GM-CSF: granulocyte-macrophage colony-stimulating factor; IL: interleukin; IFN-γ: interferon gamma; PDGF-bb: platelet-derived growth factor 2 b subunits; Ra: receptor antagonist; SIRI: systemic inflammation response index; TGF-β1: transforming growth factor-beta1; TNF-α: tumor necrosis factor-alpha; VEGF: vascular endothelial growth factor.

Table S3: Comparison of the concentrations of the test plasma biomarkers, composite inflammatory indices, MLR, NLR and PLR between primary colon vs primary rectum locations in patients with metastatic colorectal cancer.

Biomarker	Disease Site	Median	95% CI	*p*-value
CCL2	Colon	18.03	14.88 – 23.30	0.1567
	Rectum	19.56	16.86 – 26.20	
CCL3	Colon	2.21	1.79 – 2.96	0.3969
	Rectum	2.57	1.96 – 4.59	
CCL4	Colon	108.02	103.26 – 111.67	0.2301
	Rectum	99.84	90.03 – 117.23	
CCL11	Colon	58.25	51.7 – 63.15	0.3026
	Rectum	61.89	45.05 – 77.35	
CRP	Colon	3.70	2.67 – 7.58	0.6225
	Rectum	5.70	2.51 – 20.40	
CXCL10	Colon	396.03	252.05 – 520.48	0.9142
	Rectum	361.13	177.99 – 816.79	
FGF-basic	Colon	19.64	19.64 – 22.79	0.3346
	Rectum	19.64	16.15 – 22.79	
G-CSF	Colon	145.07	126.51 – 172.00	0.1961
	Rectum	172.00	135.86 – 264.50	
GM-CSF	Colon	3.40	1.84 – 4.07	0.9876
	Rectum	2.67	1.84 – 5.30	
IFN-γ	Colon	7.87	6.56 – 10.88	0.6646
	Rectum	8.31	6.56 – 10.03	
IL-1β	Colon	2.23	0.99 – 3.03	0.3800
	Rectum	2.23	0.99 – 2.83	
IL-1Ra	Colon	180.07	152.63 – 215.18	0.6432
	Rectum	229.33	180.07 – 314.34	
IL-2	Colon	0.25	0.25 – 1.31	0.6554
	Rectum	0.25	0.25 – 1.31	
IL-4	Colon	2.91	2.38 – 3.65	0.8316
	Rectum	3.28	2.38 – 4.12	
IL-5	Colon	0.95	0.93 – 14.32	0.2999
	Rectum	10.25	0.93 – 39.82	
IL-6	Colon	2.06	0.12 – 4.13	0.4453
	Rectum	2.06	0.38 – 15.67	
IL-8	Colon	9.11	5.43 – 20.89	0.1128
	Rectum	15.78	9.11 – 65.68	
IL-9	Colon	359.44	335.14 – 368.51	0.7224
	Rectum	335.14	29.71 – 363.22	
IL-10	Colon	1.97	1.29 – 3.93	0.5115
	Rectum	3.93	1.29 – 5.19	
IL-12	Colon	15.26	11.91 – 21.74	0.9876
	Rectum	21.74	8.46 – 28.02	
IL-13	Colon	6.98	5.11 – 8.32	0.7815
	Rectum	6.98	3.04 – 10.86	
IL-17A	Colon	20.35	18.68 – 23.11	0.4974
	Rectum	21.45	18.12 – 25.84	

PDGF-bb	Colon	478.70	377.14 – 554.43	0.5382
	Rectum	374.68	219.63 – 787.59	
TGF-β1	Colon	5.00	4.58 – 6.07	0.5080
	Rectum	4.83	3.30 – 6.70	
TNF-α	Colon	137.59	124.12 – 143.74	0.2543
	Rectum	132.43	104.08 – 140.67	
VEGF	Colon	9.12	9.10 – 108.19	0.2100
	Rectum	9.10	9.10 – 90.70	
AISI	Colon	463.81	179.34 – 634.8	0.3480
	Rectum	475.58	261.13 – 1155.37	
MLR	Colon	0.34	0.24 – 0.54	0.3479
	Rectum	0.42	0.25 – 0.89	
NLR	Colon	2.30	1.36 – 3.37	0.0095
	Rectum	3.72	2.78 – 7.35	
PLR	Colon	142.00	118.00 – 173.00	0.5815
	Rectum	143.00	117.00 – 201.00	
SIRI	Colon	1.29	0.74 – 2.45	0.1315
	Rectum	2.28	1.34 – 1.34	

Abbreviations: AISI: aggregate index of systemic inflammation; CCL: C-C motif ligand; CRP: C-reactive protein; CXCL: C-X-C motif chemokine ligand; FGF-basic: fibroblast growth factor-basic; G-CSF: granulocyte colony-stimulating factor; GM-CSF: granulocyte-macrophage colony-stimulating factor; IL: interleukin; IFN-γ: interferon gamma; MLR: monocyte-to lymphocyte ratio; NLR: neutrophil-to-lymphocyte ratio; PDGF-bb: platelet-derived growth factor 2 b subunits; PLR: platelet-to-lymphocyte ratio; Ra: receptor antagonist; SIRI: systemic inflammation response index; TGF-β1: transforming growth factor-beta1; TNF-α: tumor necrosis factor-alpha; VEGF: vascular endothelial growth factor.

Table S4: Comparison of the concentrations of the test plasma biomarkers, and composite inflammatory indices, MLR, NLR and PLR in metastatic colorectal cancer patients receiving first-line treatment vs. those with metastatic colorectal cancer receiving therapy in the second-line setting or beyond.

Biomarker	Line of treatment	Median	95% CI	*p*-value
CCL2	≥ line	19.18	14.47 – 23.30	0.7890
	First line	18.80	16.86 – 24.76	
CCL3	≥ line	2.50	2.13 – 3.32	0.7145
	First line	2.32	1.39 – 3.76	
CCL4	≥ line	104.19	89.07 – 109.70	0.1284
	First line	109.01	99.84 – 113.93	
CCL11	≥ line	61.50	45.05 – 71.69	0.5987
	First line	59.45	53.87 – 62.29	
CRP	≥ line	6.64	3.35 – 23.30	0.1047
	First line	3.10	2.51 – 5.79	
CXCL10	≥ line	426.43	337.98 – 531.83	0.3495
	First line	344.35	211.29 – 442.63	
FGF-basic	≥ line	22.02	16.15 – 22.79	0.4703
	First line	19.64	17.94 – 22.79	
G-CSF	≥ line	157.54	117.01 – 191.62	0.7147
	First line	151.90	135.86 – 193.78	
GM-CSF	≥ line	2.86	1.80 - 4.7	0.4351
	First line	3.22	2.67 – 4.07	
IFN-γ	≥ line	7.66	6.56 – 10.03	0.3031
	First line	8.31	7.0 – 11.74	
IL-1β	≥ line	2.13	0.99 – 2.83	0.9300
	First line	1.62	0.88 – 3.71	
IL-1Ra	≥ line	190.30	158.31 – 314.34	0.6804
	First line	192.98	169.37 – 229.33	
IL-2	≥ line	0.26	0.25 – 2.32	0.2536
	First line	0.25	0.25 – 1.31	
IL-4	≥ line	2.91	2.38 – 4.12	0.8065
	First line	3.16	2.38 – 3.88	
IL-5	≥ line	3.43	0.93 – 14.32	0.1952
	First line	10.25	0.93 – 25.70	
IL-6	≥ line	3.02	0.61 – 10.16	0.3745
	First line	1.35	0.12 – 4.13	
IL-8	≥ line	16.17	6.87 – 33.38	0.3128
	First line	10.21	5.43 – 16.04	
IL-9	≥ line	357.93	313.76 – 368.51	0.3680
	First line	336.67	332.86 – 366.24	
IL-10	≥ line	2.64	1.29 – 5.19	0.9408
	First line	1.63	1.29 – 3.93	
IL-12	≥ line	15.26	11.91 – 21.74	0.8128
	First line	21.74	8.46 – 28.02	
IL-13	≥ line	7.44	4.10 – 9.61	0.6500
	First line	6.18	3.58 – 9.40	
IL-17A	≥ line	20.90	16.99 – 24.75	0.8146

	First line	21.45	19.23 – 23.66	
PDGF-bb	≥ line	469.14	264.11 – 554.43	0.5295
	First line	449.67	312.53 – 771.64	
TGF-β1	≥ line	5.05	4.22 – 6.68	0.9194
	First line	4.92	4.50 – 6.68	
TNF-α	≥ line	137.59	119.94 – 147.83	0.5484
	First line	132.43	115.74 – 140.67	
VEGF	≥ line	9.10	9.10 – 108.19	0.4750
	First line	9.10	9.10 – 90.70	
AISI	≥ line	527.43	179.34 – 862.76	0.6935
	First line	463.81	234.53 – 634.80	
MLR	≥ line	0.54	0.27 – 0.70	0.0702
	First line	0.30	0.24 – 0.40	
NLR	≥ line	3.14	1.45 – 4.96	0.6837
	First line	2.88	1.70 – 3.72	
PLR	≥ line	141.00	118.00 – 290.00	0.4823
	First line	154.00	117.00 – 173.00	
SIRI	≥ line	2.28	0.88 – 2.87	0.2999
	First line	1.42	0.69 – 2.12	

Abbreviations: AISI: aggregate index of systemic inflammation; CCL: C-C motif ligand; CRP: C-reactive protein; CXCL: C-X-C motif chemokine ligand; FGF-basic: fibroblast growth factor-basic; G-CSF: granulocyte colony-stimulating factor; GM-CSF: granulocyte-macrophage colony-stimulating factor; IL: interleukin; IFN-γ: interferon gamma; MLR: monocyte-to lymphocyte ratio; NLR: neutrophil-to-lymphocyte ratio; PDGF-bb: platelet-derived growth factor 2 b subunits; PLR: platelet-to-lymphocyte ratio; Ra: receptor antagonist; SIRI: systemic inflammation response index; TGF-β1: transforming growth factor-beta1; TNF-α: tumor necrosis factor-alpha; VEGF: vascular endothelial growth factor.

Table S5: Comparison of the concentrations of the plasma test biomarkers, and composite inflammatory indices, MLR, NLR and PLR in metastatic colorectal cancer patients younger than 60 years of age and those 60 years of age or older.

Biomarker	Age	Median	95% CI	*p*-value
CCL2	≥ 60 years	19.94	16.86 – 24.03	0.2124
	< 60 years	17.64	14.07 – 19.56	
CCL3	≥ 60 years	2.43	1.39 – 3.97	0.9410
	< 60 years	2.36	1.79 – 3.32	
CCL4	≥ 60 years	101.86	89.07 – 111.52	0.3077
	< 60 years	107.19	100.78 – 111.67	
CCL11	≥ 60 years	54.79	41.77 – 61.89	0.1039
	< 60 years	61.42	56.98 – 69.58	
CRP	≥ 60 years	4.61	2.51 – 8.00	0.7579
	< 60 years	4.16	2.90 – 8.11	
CXCL10	≥ 60 years	354.42	211.29 – 531.83	0.9528
	< 60 years	409.47	252.05 – 512.10	
FGF-basic	≥ 60 years	20.45	19.64 – 25.70	0.8936
	< 60 years	19.64	16.15 – 22.79	
G-CSF	≥ 60 years	147.35	102.43 – 191.62	0.4332
	< 60 years	157.54	133.54 – 185.13	
GM-CSF	≥ 60 years	3.04	2.67 – 4.70	0.2972
	< 60 years	2.86	1.84 – 3.74	
IFN-γ	≥ 60 years	8.09	6.56 – 10.03	0.9881
	< 60 years	8.31	6.56 – 10.88	
IL-1β	≥ 60 years	2.73	0.99 – 3.99	0.0582
	< 60 years	1.62	0.55 – 2.43	
IL-1Ra	≥ 60 years	185.26	158.31 – 229.33	0.3329
	< 60 years	198.03	169.37 – 314.34	
IL-2	≥ 60 years	0.26	0.25 – 1.31	0.5201
	< 60 years	0.25	0.25 – 1.31	
IL-4	≥ 60 years	3.16	2.65 – 3.88	0.7973
	< 60 years	3.04	2.38 – 3.65	
IL-5	≥ 60 years	12.29	0.93 – 10.25	0.4976
	< 60 years	3.43	0.93 – 10.25	
IL-6	≥ 60 years	3.02	0.38 – 10.16	0.2680
	< 60 years	1.25	0.12 – 4.67	
IL-8	≥ 60 years	14.07	5.43 – 23.89	0.5151
	< 60 years	11.54	5.43 – 16.04	
IL-9	≥ 60 years	357.93	327.52 – 368.51	0.5510
	< 60 years	336.67	313.76 – 359.44	
IL-10	≥ 60 years	3.29	1.29 – 3.93	0.6096
	< 60 years	1.63	1.27 – 3.93	
IL-12	≥ 60 years	18.5	11.91 – 21.74	0.6213
	< 60 years	15.26	8.46 – 21.74	
IL-13	≥ 60 years	8.10	5.11 – 9.61	0.0973
	< 60 years	5.59	3.04 – 8.10	
IL-17A	≥ 60 years	18.96	16.99 – 24.75	0.3429
	< 60 years	21.45	20.35 – 23.66	
PDGF-bb	≥ 60 years	447.30	264.11 – 582.55	0.6467

	< 60 years	469.14	302.43 – 663.83	
TGF-β1	≥ 60 years	5.07	4.50 – 6.68	0.5041
	< 60 years	4.86	4.20 – 5.86	
TNF-α	≥ 60 years	136.05	115.74 – 140.67	0.8707
	< 60 years	133.47	117.85 – 140.67	
VEGF	≥ 60 years	9.11	9.10 – 70.92	0.6868
	< 60 years	9.10	9.10 – 108.19	
AISI	≥ 60 years	464.19	234.53 – 1122.85	0.5347
	< 60 years	495.62	179.34 – 716.17	
MLR	≥ 60 years	0.48	0.19 – 0.89	0.2088
	< 60 years	0.33	0.25 – 0.42	
NLR	≥ 60 years	3.31	1.36 – 6.92	0.6917
	< 60 years	2.75	1.71 – 3.68	
PLR	≥ 60 years	173.00	128.00 – 301.00	0.1651
	< 60 years	129.50	117.00 – 165.00	
SIRI	≥ 60 years	1.92	0.88 – 5.01	0.383
	< 60 years	1.68	0.74 – 2.45	

Abbreviations: AISI: aggregate index of systemic inflammation; CCL: C-C motif ligand; CRP: C-reactive protein; CXCL: C-X-C motif chemokine ligand;FGF-basic: fibroblast growth factor-basic; G-CSF: granulocyte colony-stimulating factor; GM-CSF: granulocyte-macrophage colony-stimulating factor; IL: interleukin; IFN-γ: interferon gamma; MLR: monocyte-to lymphocyte ratio; NLR: neutrophil-to-lymphocyte ratio; PDGF-bb: platelet-derived growth factor 2 b subunits; PLR: platelet-to-lymphocyte ratio; Ra: receptor antagonist; SIRI: systemic inflammation response index; TGF-β1: transforming growth factor-beta1; TNF-α: tumor necrosis factor-alpha; VEGF: vascular endothelial growth factor.

Table S6: Comparison of the concentrations of the plasma test biomarkers, and composite inflammatory indices, MLR, NLR and PLR in metastatic CRC patients by gender.

Biomarker	Gender	Median	95% CI	*p*-value
CCL2	Female	18.80	14.88 – 24.03	0.5214
	Male	19.56	15.68 – 23.30	
CCL3	Female	2.21	1.39 – 2.71	0.1165
	Male	2.57	2.13 – 4.54	
CCL4	Female	100.78	89.71 – 111.52	0.1393
	Male	107.72	102.02 – 113.93	
CCL11	Female	62.21	44.73 – 70.68	0.5403
	Male	57.93	51.70 – 63.15	
CXCL10	Female	442.63	327.57 – 677.81	0.3171
	Male	338.30	250.03 – 512.10	
FGF-basic	Female	17.94	16.15 – 22.79	0.0505
	Male	21.25	19.64 – 25.70	
G-CSF	Female	140.48	126.51 – 156.41	0.0755
	Male	172.00	140.48 – 242.09	
GM-CSF	Female	2.67	1.84 – 3.04	0.0270
	Male	3.74	2.67 – 4.70	
CRP	Female	4.16	2.61 – 7.93	0.8708
	Male	4.22	2.00 – 8.00	
IFN-γ	Female	7.44	6.56 – 8.31	0.1408
	Male	9.17	6.56 – 12.58	
IL-1β	Female	1.62	0.55 – 2.63	0.0941
	Male	2.43	1.20 – 3.99	
IL-1Ra	Female	180.07	146.83 – 229.33	0.0955
	Male	200.53	169.37 – 352.52	
IL-2	Female	0.25	0.25 – 1.31	0.3955
	Male	0.25	0.25 – 1.31	
IL-4	Female	3.16	2.38 – 3.88	0.2368
	Male	3.16	2.78 – 3.88	
IL-5	Female	0.93	0.93 – 10.25	0.0371
	Male	14.32	0.95 – 25.7	
IL-6	Female	2.45	0.12 – 7.81	0.9397
	Male	1.86	0.12 – 6.44	
IL-8	Female	7.72	5.13 – 22.64	0.2754
	Male	13.95	9.11 – 22.14	
IL-9	Female	337.43	327.52 – 363.22	0.6712
	Male	342.75	335.14 – 368.51	
IL-10	Female	1.29	1.27 – 3.93	0.0436
	Male	2.64	1.29 – 5.19	
IL-12	Female	15.26	8.46 – 21.74	0.2763
	Male	21.74	15.26 – 28.02	
IL-13	Female	5.59	3.04 – 8.75	0.1882
	Male	7.44	5.11 – 9.61	
IL-17A	Female	20.35	16.43 – 24.75	0.3306
	Male	21.45	19.23 – 24.75	
PDGF-bb	Female	374.68	219.63 – 478.70	0.0020

	Male	582.55	401.59 – 787.59	
TGF-β1	Female	4.50	3.40 – 5.86	0.0763
	Male	5.11	4.83 – 6.87	
TNF-α	Female	132.43	104.08 – 140.67	0.3237
	Male	137.59	119.94 – 143.74	
VEGF	Female	9.10	9.10 – 59.70	0.0644
	Male	47.03	9.10 – 138.95	
AISI	Female	517.71	153.01 – 750.17	0.5946
	Male	464.19	261.13 – 860.17	
MLR	Female	0.36	0.19 – 0.47	0.6048
	Male	0.36	0.25 – 0.62	
NLR	Female	3.22	1.47 – 4.02	0.8416
	Male	2.97	1.71 – 3.68	
PLR	Female	183.00	97.00 – 322.00	0.0614
	Male	128.00	118.00 – 159.00	
SIRI	Female	1.32	0.66 – 2.45	0.2675
	Male	2.05	0.92 – 3.20	

Abbreviations: AISI: aggregate index of systemic inflammation; CCL: C-C motif ligand; CRP: C-reactive protein; CXCL: C-X-C motif chemokine ligand; FGF-basic: fibroblast growth factor-basic; G-CSF: granulocyte colony-stimulating factor; GM-CSF: granulocyte-macrophage colony-stimulating factor; IL: interleukin; IFN-γ: interferon gamma; MLR: monocyte-to lymphocyte ratio; NLR: neutrophil-to-lymphocyte ratio; PDGF-bb: platelet-derived growth factor 2 b subunits; PLR: platelet-to-lymphocyte ratio; Ra: receptor antagonist; SIRI: systemic inflammation response index; TGF-β1: transforming growth factor-beta1; TNF-α: tumor necrosis factor-alpha; VEGF: vascular endothelial growth factor.
